# Detecting, extracting, and mapping of inland surface water using Landsat 8 Operational Land Imager: A case study of Pune district, India

**DOI:** 10.12688/f1000research.121740.1

**Published:** 2022-07-11

**Authors:** Rushikesh Kulkarni, Kanchan Khare, Humera Khanum

**Affiliations:** 1 Department of Civil Engineering, Symbiosis Institute of Technology, Symbiosis International (Deemed University), Lavale, Pune, Maharashtra, 412115, India

**Keywords:** Surface water, Landsat 8 OLI, MNDWI, Water index, Confusion matrix, Otsu’s Threshold

## Abstract

**Background:** Recent developments in optical satellite remote sensing have led to a new era in the detection of surface water with its changing dynamics. This study presents the creation of surface water inventory for a part of Pune district (an administrative area), in India using the Landsat 8 Operational Land Imager (OLI) and a multi spectral water indices method.

**Methods:** A total of 13 Landsat 8 OLI cloud free images were analyzed for surface water detection. Modified Normalized Difference Water Index (MNDWI) spectral index method was employed to enhance the water pixels in the image. Water and non-water areas in the map were discriminated using the threshold slicing method with a trial and error approach. The accuracy analysis based on kappa coefficient and percentage of the correctly classified pixels was presented by comparing MNDWI maps with corresponding Joint Research Centre (JRC) Global Surface Water Explorer (GSWE) images. The changes in the surface area of eight freshwater reservoirs within the study area (Bhama Askhed, Bhatghar, Chaskaman, Khadakwasala, Mulashi, Panshet, Shivrata, and Varasgaon) for the year 2016 were analyzed and compared to GSWE time series water databases for accuracy assessment. The annual water occurrence map with percentage water occurrence on a yearly basis was also prepared.

**Results:** The kappa coefficient agreement between MNDWI images and GSWE images is in the range of 0.56 to 0.96 with an average agreement of 0.82 indicating a strong level of agreement.

**Conclusions:** MNDWI is easy to implement and is a sufficiently accurate method to separate water bodies from satellite images. The accuracy of the result depends on the clarity of image and selection of an optimum threshold method. The resulting accuracy and performance of the proposed algorithm will improve with implementation of automatic threshold selection methods and comparative studies for other spectral indices methods.

## Introduction

By 2030, the world is anticipated to face a 40 percent water crisis as a result of urbanization, strain on food production, population increase, and increasing demand from the industrial, domestic, and energy sectors ^1, 2^. The current water crises are a result of poor water management and not because of adequacy of water. Sustainability of water can be maintained with proper governance and appropriate water management practices ^3^. Inland water bodies are critical for the survival of all forms of life and need to be monitored frequently ^4^. The regular monitoring and mapping of surface water is of paramount importance for better water resource management ^5^.

 Surface water bodies are dynamic in nature as they shrink and expand with the course of time ^6^. Remote sensing is an effective and low cost alternative to conventional in-situ water quality monitoring systems ^7, 8^. Recent advancements in optical remote sensing have catapulted the field of surface water sensing into a new era. It has been used in water resource assessment and management in the last four decades ^9– 19^. These applications involve delineation of surface water and assessing water quality using thematic information extraction techniques ^20^.

Both active and passive remote sensing have been used to detect and extract surface water bodies ^9^. Optical remote sensing of water is based on the difference between spectral reflectance of land and water. Water absorbs most of the energy beyond near-infrared wavelength whereas other land features have higher reflectance in those wavelengths. Thus, water appears as a dark spot in near-infrared and higher wavelength bands of multispectral images which makes it easy to differentiate water from non-water topographic features. The accuracy depends on the weather conditions during the image captured and topography of the study area. Microwave is very effective during the presence of clouds, rain fields, the presence of water vapor and aerosols. Microwave remote sensing of surface water is based on the backscattering properties of water in the microwaves region. The surface of water behaves as a specular reflector for incident microwaves, and responds with no or very low signal returns. Thus, water is easily distinguished from other topographic features in and around the area.

Applications of remote sensing for various purposes of water resource management were proposed for the first time for surface water bodies in India ^21^. Moderate Resolution Imaging Spectroradiometer (MODIS), a coarse resolution but high temporal resolution sensor have been successfully applied to near-real time surface water monitoring ^22– 25^. The Visible Infrared Imaging Radiometer Suite onboard Suomi National Polar-orbiting Partnership (Suomi NPP-VIIRS) has been tested for detecting surface water ^26^. The low resolution satellites have major limitations in observing small water bodies. Cloud cover is another issue often associated with low resolution satellites. Landsat is the most successful series of satellites in monitoring surface water bodies in the history of remote sensing ^9^. Landsat 8 Operational Land Imager (OLI) is the most recent satellite in the Landsat series and is widely used in detecting surface water bodies ^11– 19^. Systeme Probatoire d’Observation dela Tarre (SPOT), which has an improved spatial resolution of 10 m but that is not available free of cost, is used in a few studies for applications of surface water ^27, 28^. The MultiSpectral Instrument (MSI) onboard Sentinel-2 with spatial resolutions of 10m and a revisit time of 10 days at equator and five days with two satellites under cloud free conditions is used to detect surface water ^29– 31^. Many works specifically reviewed recent accomplishments, current status, and limitations in this domain, including geographic scale, integration with in-situ hydrological data, cloud obscuration, and other land feature classes. ^7– 9, 32– 34^.

Water extraction methods using remote sensing have been pursued by advancing sensors and through the development of various methods like thematic classifications, Linear Unmixing, single band thresholding and multispectral water indices ^5, 9, 20, 35^. The method selection from the list above is based on the scale of the water map and the proportion of water bodies to non-water area in the image.

The method of extracting features of surface water using water indices remains popular because it is simple to implement, has a relatively fast processing time, and produces fairly accurate results ^36, 37^. These methods have produced excellent results when used with Landsat imagery ^11, 38, 39^. Surface water features can be distinguished using a variety of water indexes, including Normalized Difference Water Index (NDWI), Modified Normalized Difference Water Index (MNDWI), and Automated Water Extraction Index (AWEI). NDWI uses the green and near infrared (NIR) band to delineate water features in the image ^40^. Nevertheless, The NDWI is unable to suppress the built-up land signal which make it difficult to differentiate water and built up area effectively ^20^. MNDWI is a modified version of NDWI that uses green and short-wave infrared (SWIR) bands to overcome limitations encountered by NDWI for extracting water using space observation. AWEInsh, an automated water extraction index with no shadow, and AWEIsh, an automated water extraction index with a shadow, are two more additional water indices that have been proposed for water delineation ^41^.

Selecting an optimum threshold to discriminate water accurately from land is a challenging and complex task ^41^. Ideally, spectral index images assign higher values to water and negative values to the non-water pixels. Thus, zero is ideally a reference value to separate water and non-water pixels. However, in practice most of the times the threshold values are non-zero. In this study automated Otsu’s threshold method and trial-and error methods are used to estimate optimum threshold values ^42, 43^.

Pekel (2016) presented a comprehensive validation protocol for accuracy analysis of the Global Surface Water Explorer (GSWE) dataset ^44^. This dataset has been validated using the total 40,124 control points distributed geographically and temporarily all across the globe. GSWE is produced from Landsat imagery at the global scale over the past 32 years.

In this study, we demonstrated a case study of Pune district for detection and mapping of surface water using the MNDWI spectral index method. The objective of the study is evaluation of the MNDWI water indices method for extraction of surface water from Landsat 8 OLI images having multiple water bodies located spatially apart. The time series surface water map is prepared for the entire year using the cloud free images. The surface water maps so developed are assessed for accuracy by comparing them with a reference surface water map prepared by The European Commission’s Joint Research Centre in the framework of the Copernicus Programme.

## Methods

The processes followed for achieving the objectives of study are represented in Figure 2. In summary, the process starts with downloading the cloud free Landsat 8 OLI of the study area (see *Underlying data*) and carrying out initially radiometric and geometric corrections, and cloud detection. In the next step MNDWI spectral indices were calculated from the Landsat images in order to enhance all water pixels in the image. Water bodies were extracted using the threshold slicing method using Otsu and trial and error method. Finally, the images were assessed for accuracy by comparing with a reference image. All processes followed are explained in the separate subsections below.

### Study area

**Figure 1.**
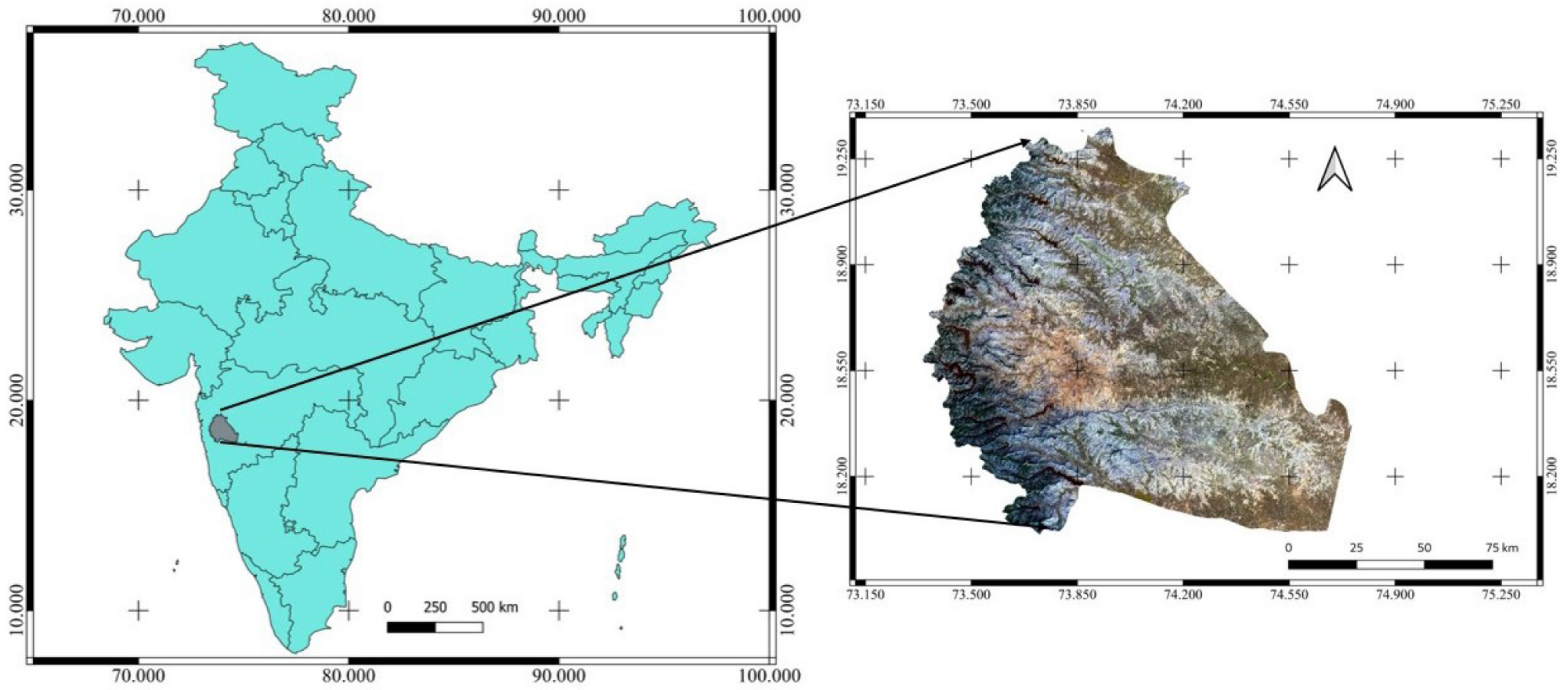
Study area location and extent. Landsat 8 image courtesy of the U.S. Geological Survey.

The study area for development of surface water maps is between 18.01 N and 19.36 N and 73.34 E and 74.76 E which is a portion of the Pune district administrative boundary in India. Pune lies on the west of the Deccan plateau ( Figure 1), at an altitude of 560 m (1,840 ft) above mean sea level. It is located in western Maharashtra along the foothills of the Sahyadri Mountains ^45^.

**Figure 2.**
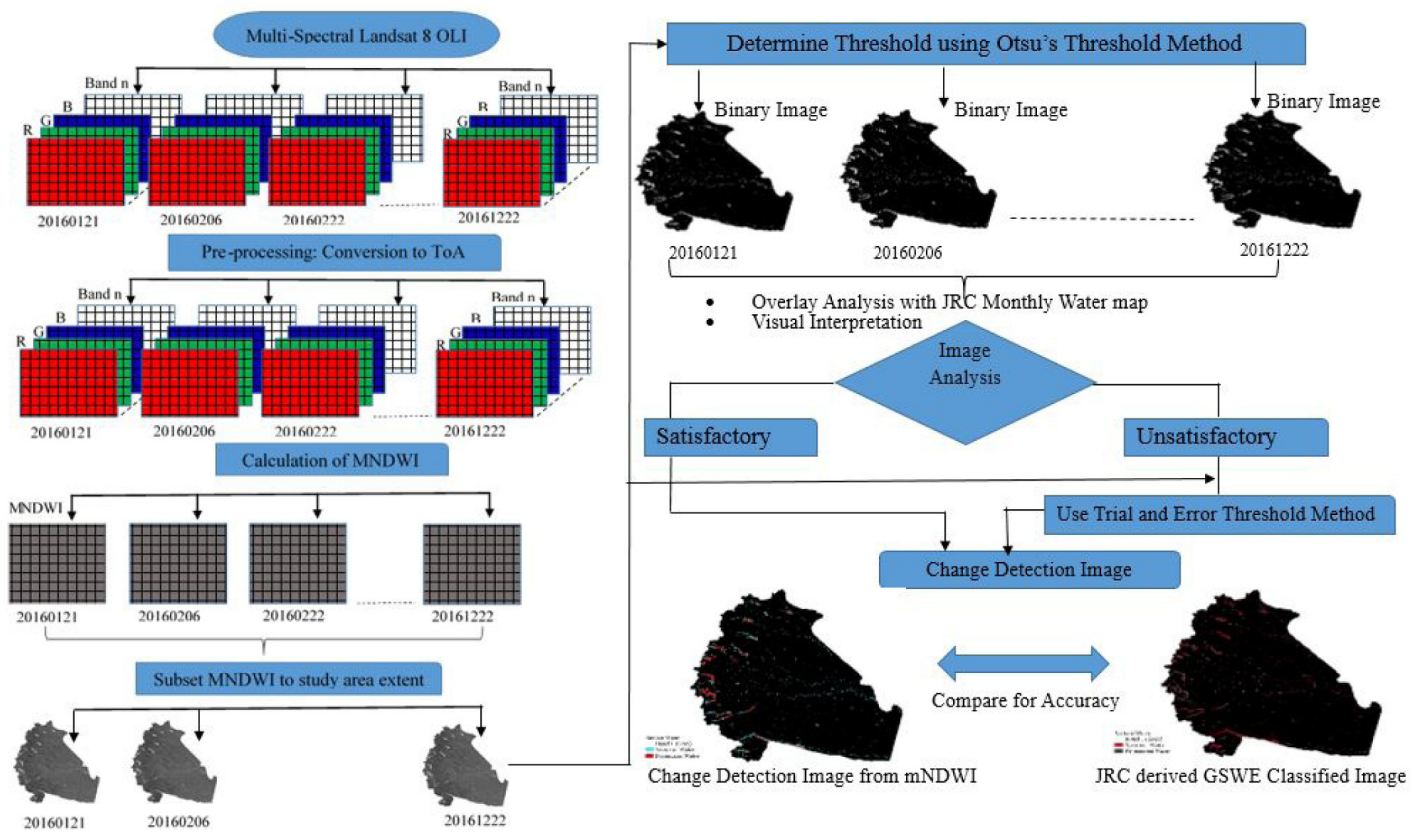
Flow chart of the methodology used. OLI, Operational Land Imager; ToA, Top of Atmosphere; MNDWI, Modified Normalized Difference Water Index; JRC, Joint Research Centre; GSWE, Global Surface Water Explorer. Landsat 8 images courtesy of the U.S. Geological Survey.

This area is chosen for two reasons. First, it has heterogeneous topography comprising hills on the western side and plain terrain on the eastern side of the district. It covers all the types of surface water bodies, such as rivers, lakes, and reservoirs. Secondly, the authors are from this region, which provides prior knowledge and understanding of the selected area.

According to the 2011 Census of India, Pune district had a total population of 9.429 million, meaning it lies within India’s top ten most populated cities ^45^. The monsoon season in Pune is observed from June to September, and the cloud presence makes it challenging for water extraction from remote sensing. Therefore, surface water extraction analysis were performed for the non- rainy season.

### Data: Landsat 8 OLI

Landsat is one of the best known satellite series in history. Since the first mission was initiated in 1972, medium-resolution photographs have been consistently supplied for almost 50 years now. Landsat 8 OLI images have a moderate spatial resolution (30 m), spectral resolution (eleven bands) and temporal resolution of 16 days. Launched on 11 February 2013, Landsat 8 is the most recent Landsat satellite. It is fitted with an enhanced ”Operational Land Imager (OLI)” sensor with the addition of two thermal Infra- Red sensors (TIRS). The Landsat 8 OLI sensor has a better rate of classification because it provides improved 12-bit data quantization compared to 8-bit quantization of other sensors. A total of 13 Cloud free Landsat 8 OLI images were selected and downloaded from the US Geological Survey (USGS) earth explorer (see *Underlying data*) for the study area during the year 2016 ( Table 1). All images contained 1 – 7 bands with 30 m resolution. The quality of the image is ensured with the information in pixel- qa product downloaded along with the Landsat 8 image. This product provided the information about cloud and cloud confidence and cloud shadow. More information about the quality evaluation can be found here.

**Table 1.** Landsat 8 Operational Land Imager (OLI) images downloaded and analysed in this study.

Sr.No	Landsat 8 OLI	Date	Path/Row
1.	LC08_L2SP_147047_20160121_20200907_02_T1	21 January 2016	147/47
2.	LC08_L2SP_147047_20160206_20200907_02_T1	06 February 2016	147/47
3.	LC08_L2SP_147047_20160222_20200907_02_T1	22 February 2016	147/47
4.	LC08_L2SP_147047_20160309_20200907_02_T1	09 March 2016	147/47
5.	LC08_L2SP_147047_20160325_20200907_02_T1	25 March 2016	147/47
6.	LC08_L2SP_147047_20160410_20200907_02_T1	10 April 2016	147/47
7.	LC08_L2SP_147047_20160426_20200907_02_T1	26 April 2016	147/47
8.	LC08_L2SP_147047_20160512_20200907_02_T1	12 May 2016	147/47
9.	LC08_L2SP_147047_20161019_20200905_02_T1	19 October 2016	147/47
10.	LC08_L2SP_147047_20161104_20200905_02_T1	04 November 2016	147/47
11.	LC08_L2SP_147047_20161120_20200905_02_T1	20 November 2016	147/47
12.	LC08_L2SP_147047_20161206_20200905_02_T1	06 December 2016	147/47
13	LC08_L2SP_147047_20161222_20200905_02_T1	22 December 2016	147/47

### Image pre-processing

**Figure 3.**
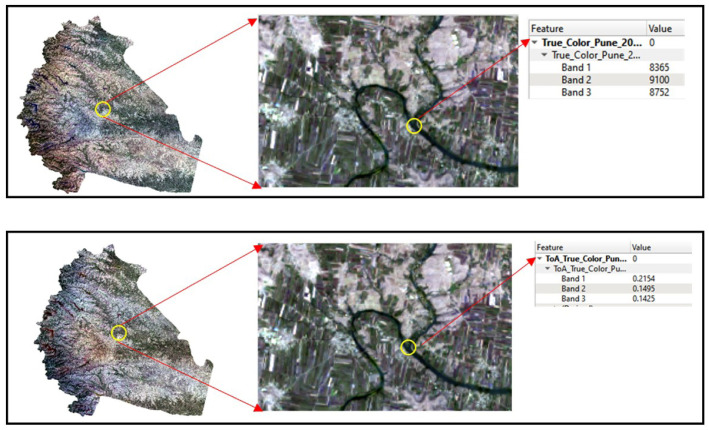
Raw Landsat 8 image of 6th February 2016 representing conversion of Digital Numbers (DN) to Top of Atmosphere (ToA) Reflectance. Landsat 8 images courtesy of the U.S. Geological Survey.

The first step in the pre-processing is to transform raw digital numbers (DN) to Top of Atmosphere (ToA) reflectance ( Figure 3). The pre-processing is performed using Semi-Automatic Classification Plugin of QGIS (3.22.3) open source software (please note that all pre-processing of the raw images, calculation of MNDWI, cropping of images and thresholding using trial and error presented in the methods is performed using QGIS open source software). Landsat Collections Level-1 data can be re-scaled to top of atmosphere (ToA) reflectance using radiometric re-scaling coefficients (see *Underlying data* ^46^). DN is a mathematical value of spectral data and not physical measured data so it is to be converted to ToA. DN can be converted to ToA reflectance using the re-scaling coefficients in the MTL using ( Equation 1):

$$\rho^{\prime }=M_{p}*Q_{cal}+A_{p}\;(1)$$

Where *M _p_* and *A _p_* are the metadata defined multiplicative and additive band specific re-scaling factors, respectively. The quantized and calibrated standard product pixel values are denoted by *Q _cal_*. It is not corrected for the sun angle, which results in the ToA reflectance as ( *ρλ*),

$$\rho \lambda =\frac{\rho \lambda^{\prime }}{cos\theta_{SZ}}=\frac{\rho \lambda^{\prime }}{sin\theta_{SE}}\;(2)$$

where *θ _SE_* is the local sun elevation angle as specified in the metadata and *θ _SZ_* denotes the local solar zenith angle as calculated using (90 ^0^ - *θ _SE_*).

### MNDWI spectral index

MNDWI were initially calculated to enhance the difference between water and non-water areas. Surface water bodies tend to have positive values in MNDWI images, whereas, soil, vegetation and built up areas are expected to represent negative values ^20^. Xu (2006) proposed that the Short-wave Infrared (SWIR) band can reflect subtle features of water more effectively compared to Near Infrared (NIR) band and substituted SWIR for NIR band in NDWI ^20^. The MNDWI method has been commonly used and is a powerful index that can extract water bodies. It is expressed by an Equation (3).

$$mNDWI=\frac{\rho 3-\rho 7}{\rho 3+\rho 7}\;(3)$$

Where the subscript *ρ* denotes the surface reflectance value computed from bands 3 (green), and 7 (visible) (SWIR-2).

**Figure 4.**
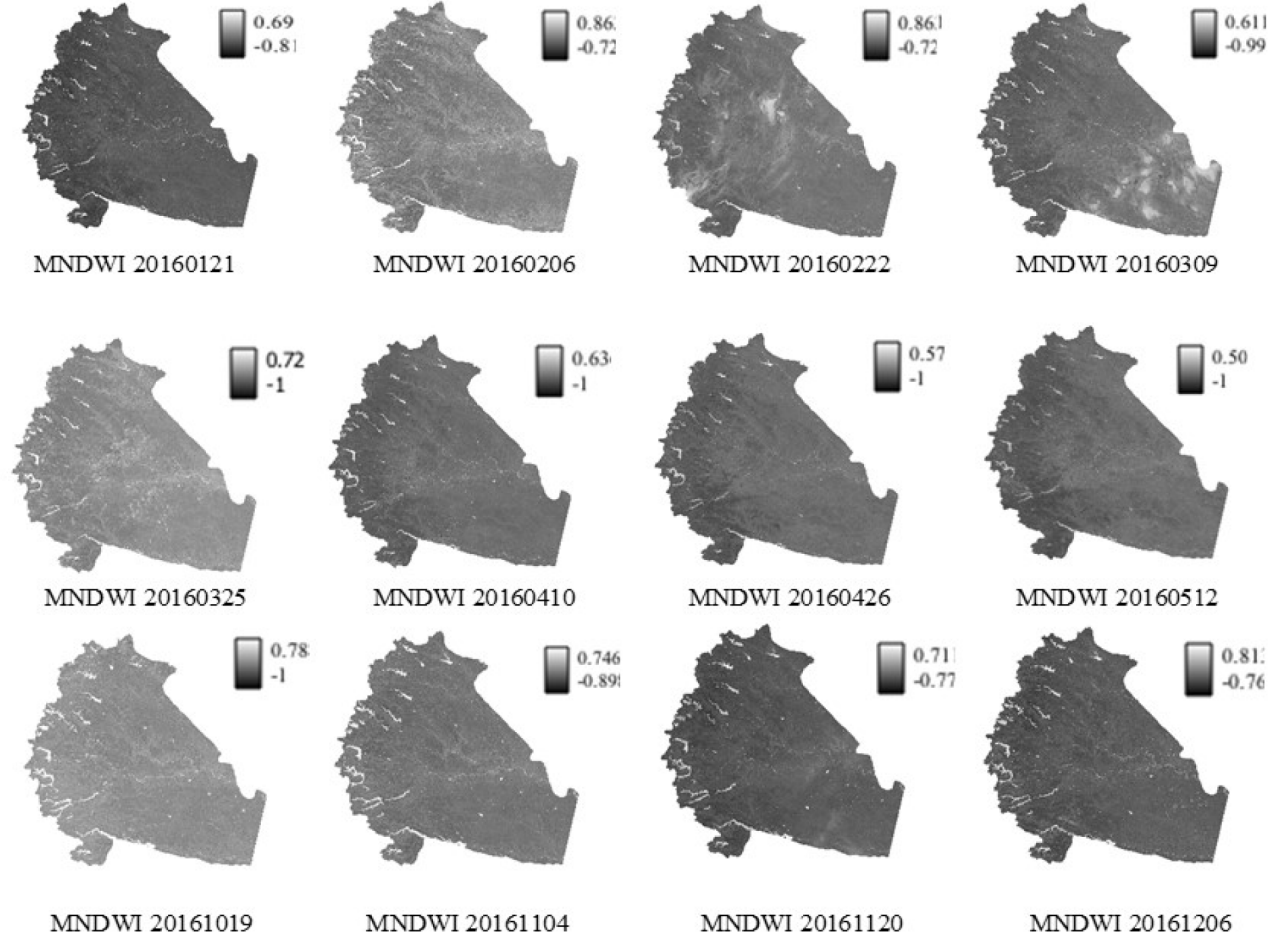
Time series Modified Normalized Difference Water Index (MNDWI) derived from Landsat 8 Operational Land Imager (OLI). Landsat 8 image courtesy of the U.S. Geological Survey.

This study restricts the surface water bodies mapping to the Pune district administrative area. The MNDWI images are subsetted using the shapefile to remain focused on the surface water bodies in the study area ( Figure 4). All the image processing algorithms mentioned in this section are performed using QGIS (3.22.3) open source software.

### Otsu threshold

Otsu’s threshold is a simple and effective approach for determining the optimum threshold ^24^. In Otsu’s threshold method all the pixels of the image are separated by discriminant creation so as to maximize the separability of two resultant classes, including object and background. In an ideal case the histogram has two distinct peaks representing object and background so that the threshold can be chosen at the bottom between two peaks. Therefore, it is essential to calculate the grey-level histogram for the whole image before calculating the optimal threshold. Google Earth Engine (GEE) open source cloud platform was used to run the Otsu’s threshold algorithm. The link to the Java code of the algorithm in GEE can be found in *Extended data* ^47^. The MNDWI image was an input to the GEE platform, and the expected result is a binary water and no- water image. We encountered a problem of the maximum number of pixels in an image exceeding the permissible number of pixels while running Otsu’s algorithm on GEE platform. The polygon area of the study region is split into two small polygons in the form of shape-file. We then estimated separately the threshold for the small areas which were then combined in the freely available QGIS (version 3.22.3) to get the surface map. Although, Otsu is described as simple approach in the literature, its success depends on many parameters like grey scale distribution, proportion of the two classes, the nature of images and many others.

### Trial and error method of threshold

**Figure 5.**
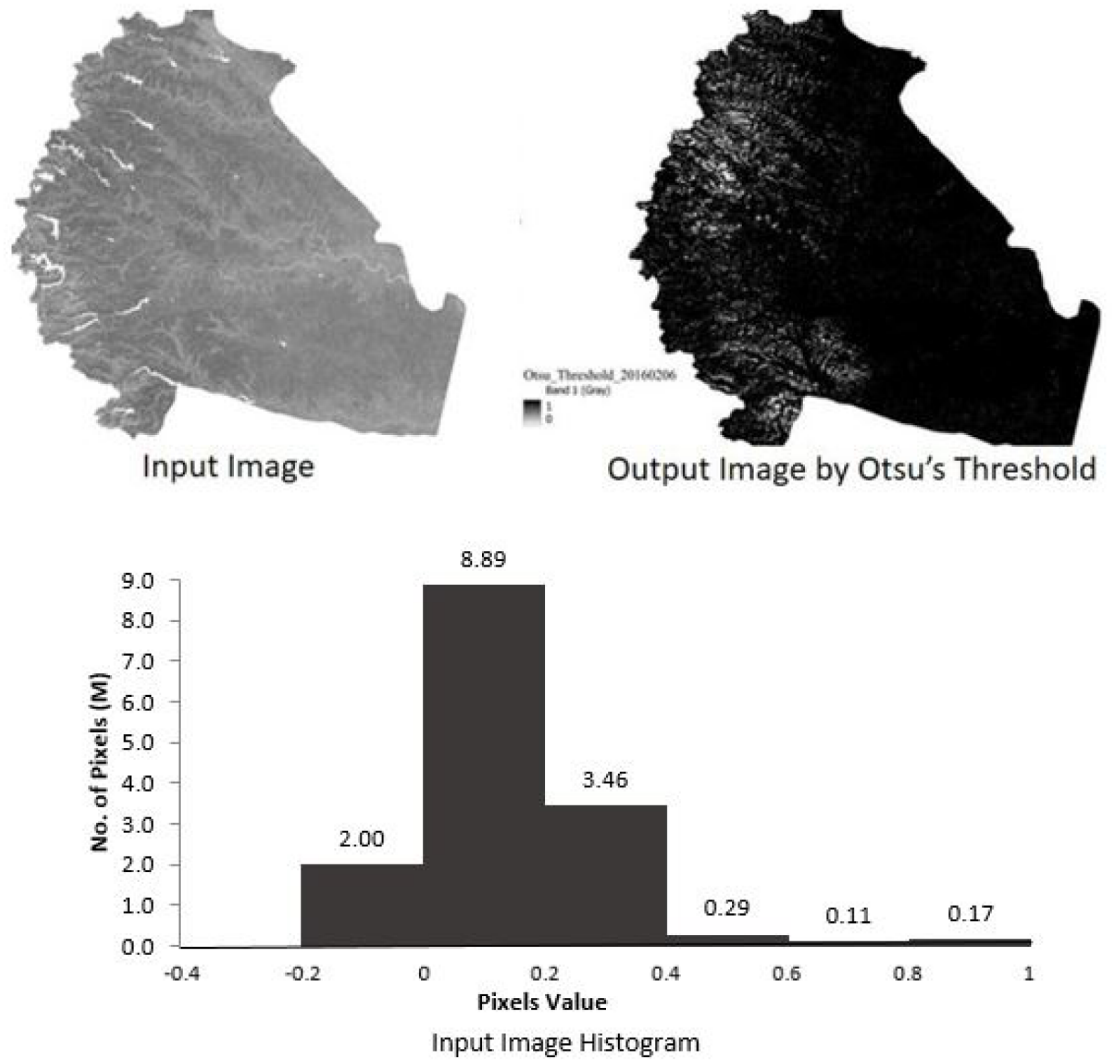
Result of Otsu’s threshold for Modified Normalized Difference Water Index (MNDWI) image on 6th February 2016. Landsat 8 image courtesy of the U.S. Geological Survey.

The optimal threshold estimated using the Otsu threshold method did not work satisfactorily and the image obtained encountered ‘salt-pepper error’ ( Figure 5). The alternative, iterative trial and error method based on visual interpretation was used in this study to find the optimal threshold to discriminate water and non-water from the MNDWI image. An initial threshold of 0 was applied to all the water index images to judge classification status by visual interpretation. Based on the initial screening, the threshold is adjusted to get higher kappa values. The optimal threshold so selected was used to generate surface water map. This water map was analyzed for classification accuracy compared with the JRC monthly map, available in *Underlying data* ^48^.

### Mountain shadow depth

The major limitations of MNDWI is that, it easily misclassifies shadow and dark roads as water bodies ^23^. In this work, the terrain shading plugin of QGIS was used to simulate mountain shadows derived from the digital elevation model (DEM) and the position of the sun. The position of the sun is available in the Metadata file downloaded with the other spectral bands from USGS. Considering the error of overestimate of water bodies, all common pixels classified as water and also in the region of shadow depth, are removed by raster calculator using logical equation.

### Yearly water occurrence map

The binary images of surface water during the non-rainy season were developed for the year 2016, which depicts water and non-water areas. These monthly water images were added by raster calculator and percentage occurrence of water for a year were estimated. The frequency with which pixels classified as water, are represented as percentages of occurrence.

### Accuracy assessment

Surface water maps produced by water index method over time should be verified for accuracy as the images are historic and because surface water changes with time. There is no physically measured database of water surface area at that point of time to compare. Thus, the obtained MNDWI water maps are compared with JRC GSWE surface water maps of the corresponding months (see *Underlying data* ^48^).

The accuracy analysis is performed using a confusion matrix method developed by comparison between reference images and the indexed-based surface water map ^24, 32^. It represents the correspondence between the resulting image of classification and the reference image. The method is well documented and illustrated in many references to calculate the confusion matrix parameters ^18, 33^. Those articles are referred to and followed in this study. The Producer’s Accuracy (PA), User’s Accuracy (UA) and Overall Accuracy (OA) represent the correctness classification of surface water images and are expressed in percentage. These calculations do not consider the agreement between data-sets that occur by chance alone. Hence, the kappa coefficient tool to control the random agreement factor, is often used. Kappa coefficient ranges from -1 to + 1, where 0 represents agreement occurring by chance and 1 represents perfect agreement.

## Results and discussion

After the radiometric corrections and the creation of the subset to study area extent, all images are processed (using QGIS 3.22.3) for derivation of water indices (see Figure 4). The figures use the YYYYMMDD format to express dates corresponding to the Landsat image. The MNDWI images clearly visualizes enhancement in open surface water features. Surface water features appear bright compared to other non-water features. The western side of the study region is mountainous, which is where the majority of surface water bodies are located. Among all the derived images the MNDWI index is in the range of -1 to 0.86. The brightness variation is observed because of the topographic corrections characterized by undulating and complex terrain. MNDWI images derived on 22nd February 2016 and 9th March 2016 indicate cloud cover of 23.09 % and 9.83 %, respectively.

The water indices images are processed for separation of water and non-water regions using a threshold slicing method. The Otsu’s threshold method is evaluated for threshold slicing for all available images. The derived binary classified image, consisting of water and non-water pixels, is compared for accuracy to the JRC GSWE water map for the corresponding month using a confusion matrix. The threshold value and confusion matrix parameters for each image are presented in Table 2. The result obtained using the Otsu’s threshold method were unsatisfactory and the model failed to produce decent results (see Figure 5). The sample calculation for clear and cloud free image captured on 6th February 2016 have been presented for discussion. The results for the rest of the images are similar. The input MNDWI image, corresponding grey scale histogram and output surface water image from Otsu’s threshold are presented in the Figure 5. MNDWI images have the range of values(-1 to 0.86), resulting in the formation of multi-modal histograms. The output image is a result of ’salt-and-pepper’ noise. The threshold value obtained by Otsu method for this image is -0.04. The threshold divides images into two classes: pixel values less than -0.04 and pixel values greater than -0.04.

**Table 2.** Accuracy assessment for Otsu method and trial and error threshold method for extraction of surface water bodies.

Dates (YYYYMMDD)	Otsu Threshold Method					Trial-Error Method				
	Thsd.	PA	UA	OA	Kappa	Thsd.	PA	UA	OA	Kappa
20160121	-0.28	96.76	3.88	48.98	0.035	0.15	86.31	91.91	99.55	0.89
20160206	-0.04	100	42.46	44.38	0.05	0.46	94.4	70.87	99.15	0.81
20160222	-0.24	97.63	3.58	69.65	0.05	0.46	74.83	45.54	97.71	0.56
20160309	-0.08	99.10	2.28	55.85	0.02	0.18	100	92.86	99.94	0.96
20160410	-0.30	98.11	2.93	69.39	0.04	0.20	81.5	88.72	99.73	0.85
20160512	-0.38	97.43	2.21	46.96	0.02	0.12	45.55	94.39	99.58	0.61
20161019	-0.05	99.52	2.99	49.53	0.03	0.34	94.31	64.99	98.53	0.76
20161104	-0.14	99.76	5.00	47.87	0.05	0.31	94.53	88.13	99.70	0.91
20161120	-0.25	99.97	58.35	70.36	0.45	0.17	87.66	92.75	99.47	0.90
20161206	-0.20	98.90	5.09	70.02	0.07	0.21	90.47	88.92	99.44	0.89

Thsd, Threshold; PA, Producer’s Accuracy; UA, User’s Accuracy; OA, Overall Accuracy.

Forward class pixels with values less than the cutoff have a majority of non-water characteristics. This portion of the binary image appears as ’salt’. The pixels with values more than threshold includes maximum pixels with water and other features as well. Background classes have a large number of pixels compared to forward classes. However, it has been demonstrated that this method produces unstable results when the proportions of water and non-water pixels are not equal and do not form a bimodal histogram ^34^. Data transmission error while calculating the threshold in Otsu’s algorithm could be another reason for the unsatisfactory result.

The iterative trial and error method was used as an alternative to the Otsu method ( Figure 7). The optimum threshold value was determined for each image. The result classified binary image is compared with the reference image. The confusion matrix parameters are performed for all images of MNDWI derived by both Otsu’s Method and trial and error method ( Table 2). The threshold by the Otsu’s method is less than zero, whereas, the threshold by trial and error method are all positive values. Based on the classification point of view, the producer’s accuracy (PA) represents error of omission. Otsu’s method analyses the gray scale histogram and classifies more pixels as water. PA of surface water map derived using the Otsu’s method and trial and error method, indicate the proportion of the GSWE image that is classified as water. More pixels of the water class reduces the percentage of omission in comparison with the GSWE water map. Both methods result in high PA for classification. UA of MNDWI surface water map derived using the Otsu’s method and trial and error method, represent the probability that pixels classified as water actually represent water in the GSWE data. UA measures error of commission, which represents the number of pixels assigned to the incorrect class. The Otsu’s threshold method classified all pixels with a gray scale level more than the respective threshold as water. This class has other topographic features along with water. Thus, the error of commission obtained is high for the MNDWI surface water maps derived by the Otsu’s method. However, iterative trial-and-error method performed better in terms of UA for surface water mapping. The minimum threshold value 0.12 is applied to the image captured in May and maximum threshold value 0.46 is applied to images acquired in February. The threshold values depend on the MNDWI range of the image. The MNDWI index observed is higher in February and lower in May among all the images of the study area. The summer season in the study area peaks around May during which the majority of surface water bodies shrink and have less water availability (see Table 3). The reduced amount of water causes turbidity and exposes silt affecting the satellite reflectance, resulting in a reduction in the number of classified water bodies. This is the reason for less MNDWI index during the summer season, between April and May. The monsoon season is observed during June to September in the study area during which the reservoirs are in full water condition (i.e. the reservoir is filled with maximum capacity of water). However the presence of cloud limits the availability of clear images. The images of immediate post monsoon season are observed with high (>0.7) MNDWI values. The non-water area has a wetness factor, which is why the lower MNDWI index has been raised from -1 to -0.7. The reservoirs with full water conditions can be easily visualized and delineated by the MNDWI method.

**Table 3.** Comparison of surface area of distinct reservoirs calculated by Modified Normalized Difference Water Index (MNDWI) and Global Surface Water Explorer (GSWE) datasets.

Dates	Methods	Bhama Askhed	Bhatghar	Chaskaman	Mulashi	Panshet	Shivrata	Varasgaon
Jan 16	MNDWI	12.22	16.26	9.87	34.02	10.73	10.20	6.14
	GSWE	14.73	20.20	11.60	36.06	11.59	10.82	7.63
Feb 16	MNDWI	12.33	15.12	10.33	34.80	11.15	10.00	6.15
	GSWE	12.52	15.52	10.27	34.51	11.12	9.95	6.17
Mar 16	MNDWI	9.82	13.93	6.46	30.16	8.71	6.48	4.43
	GSWE	11.90	15.23	8.11	32.90	10.04	8.18	5.27
Apr 16	MNDWI	8.22	11.85	5.60	29.23	6.53	5.27	4.61
	GSWE	10.01	13.29	6.17	30.94	8.85	5.82	5.10
May 16	MNDWI	1.10	9.07	4.16	15.48	3.68	0.22	2.06
	GSWE	6.76	10.42	5.64	27.57	6.53	4.15	4.36
Oct 16	MNDWI	15.96	34.66	17.06	43.21	14.61	12.45	19.61
	GSWE	14.08	30.59	15.06	38.01	12.71	11.75	17.92
Nov 16	MNDWI	14.71	33.00	16.23	40.04	13.38	12.14	19.44
	GSWE	14.53	31.51	15.34	38.59	13.35	11.80	18.36
Dec 16	MNDWI	13.74	28.93	14.20	38.84	13.47	12.09	14.15
	GSWE	14.20	30.97	14.42	38.50	13.43	11.81	18.19

### Mountain shadow

**Figure 6.**
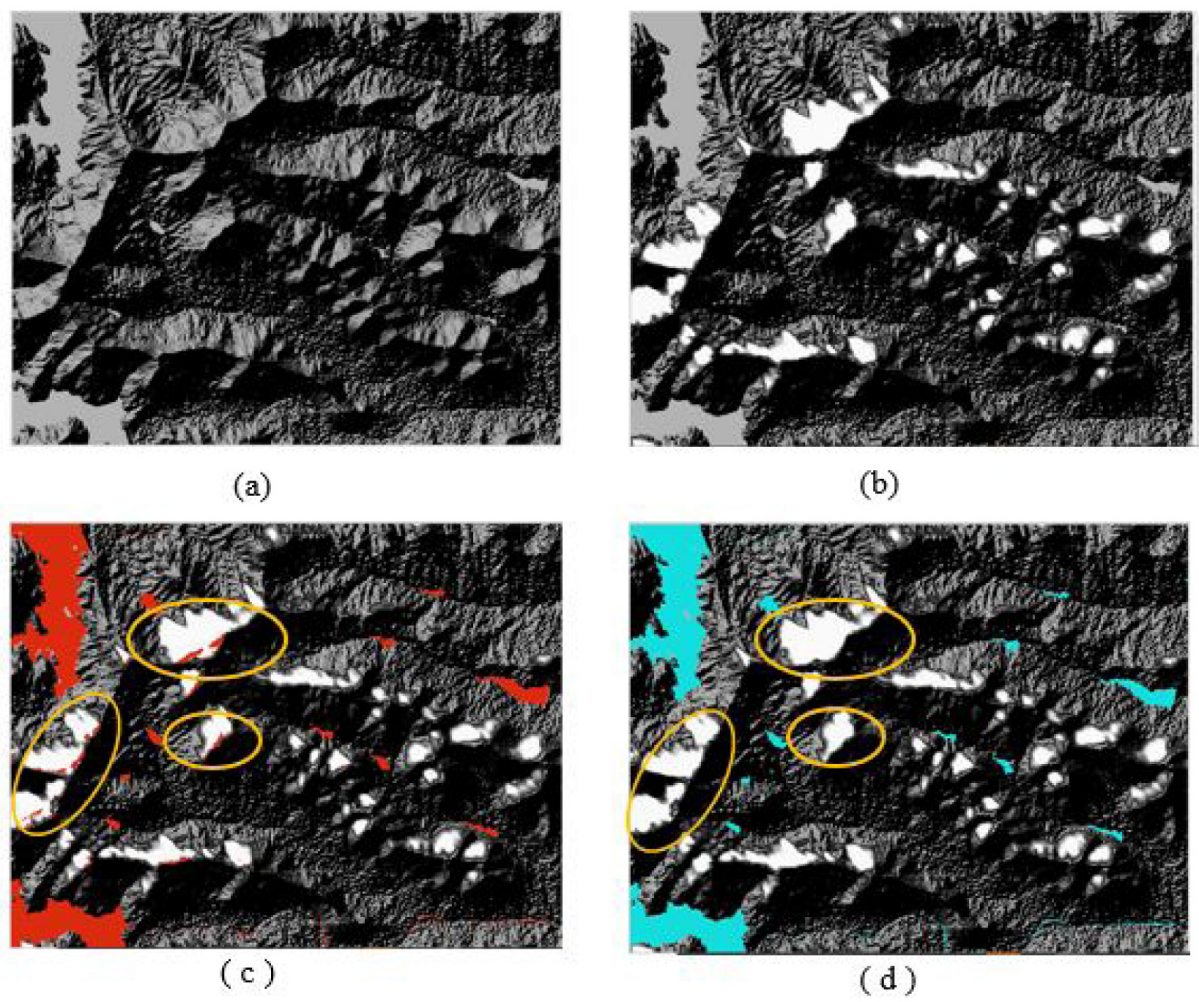
( **a**) Digital Elevation Model (DEM) of Pune. ( **b**) Mountain shadow depth derived from DEM. ( **c**) Misclassified mountain shadows as water (red area is water and white area is mountain shadow). ( **d**) Integrating shadow depth with binary Modified Normalized Difference Water Index (MNDWI) image to remove misclassified water. Landsat 8 image courtesy of the U.S. Geological Survey.

Mountain shadows have sometimes been misclassified as water. The DEM have been used to detect the misclassified portion of the surface water by mountain shadow noise ( Figure 6 (a)). The mountain shadow depth in relation to sun elevation angle and DEM have been derived, and the mountain shadow depth can be seen as the white spots in the Figure 6 (b). The surface water derived by trial and error method to MNDWI spectral image is the overlay with the mountain shadow depth in Figure 6 (c). This misclassification is removed by applying logical operation ( Figure 6 (d)). Surface water bodies located towards the western hilly regions of the study area are extracted successfully ( Figure 8). The MNDWI result is presented in Table 2, and all are followed by shadow depth analysis for removal of misclassification of water bodies.

**Figure 7.**
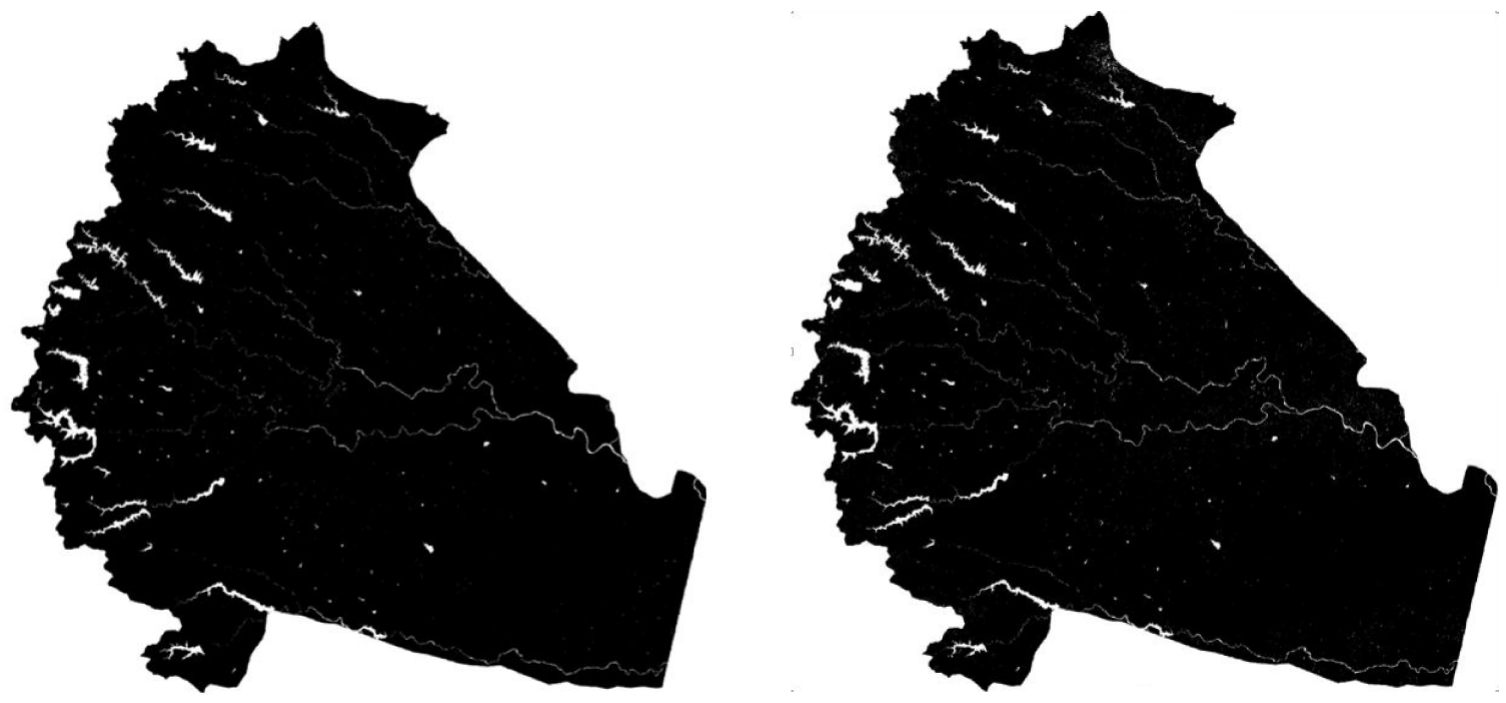
Sample image of trial and error threshold images on the date 21st January 2016 and 6th February 2016. Landsat 8 image courtesy of the U.S. Geological Survey.

### Annual surface water occurrence map

**Figure 8.**
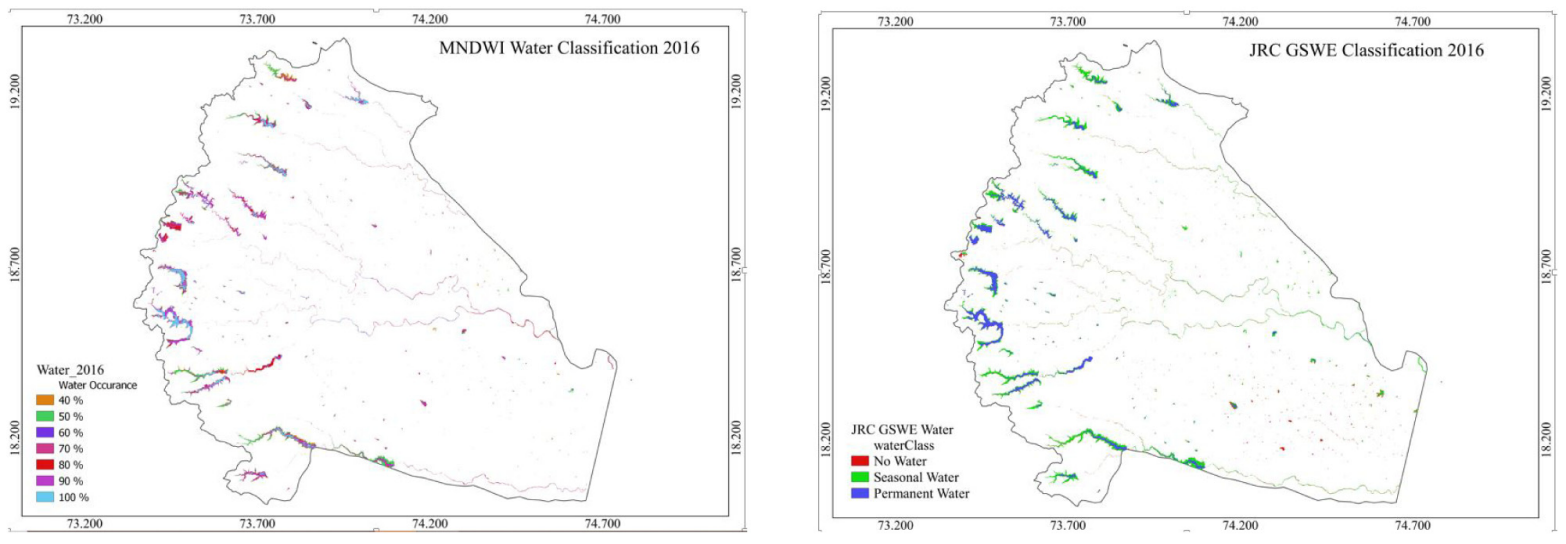
Annual water occurrence by Modified Normalized Difference Water Index (MNDWI) method and Joint Research Centre Global Surface Water Explorer.

**Figure 9.**
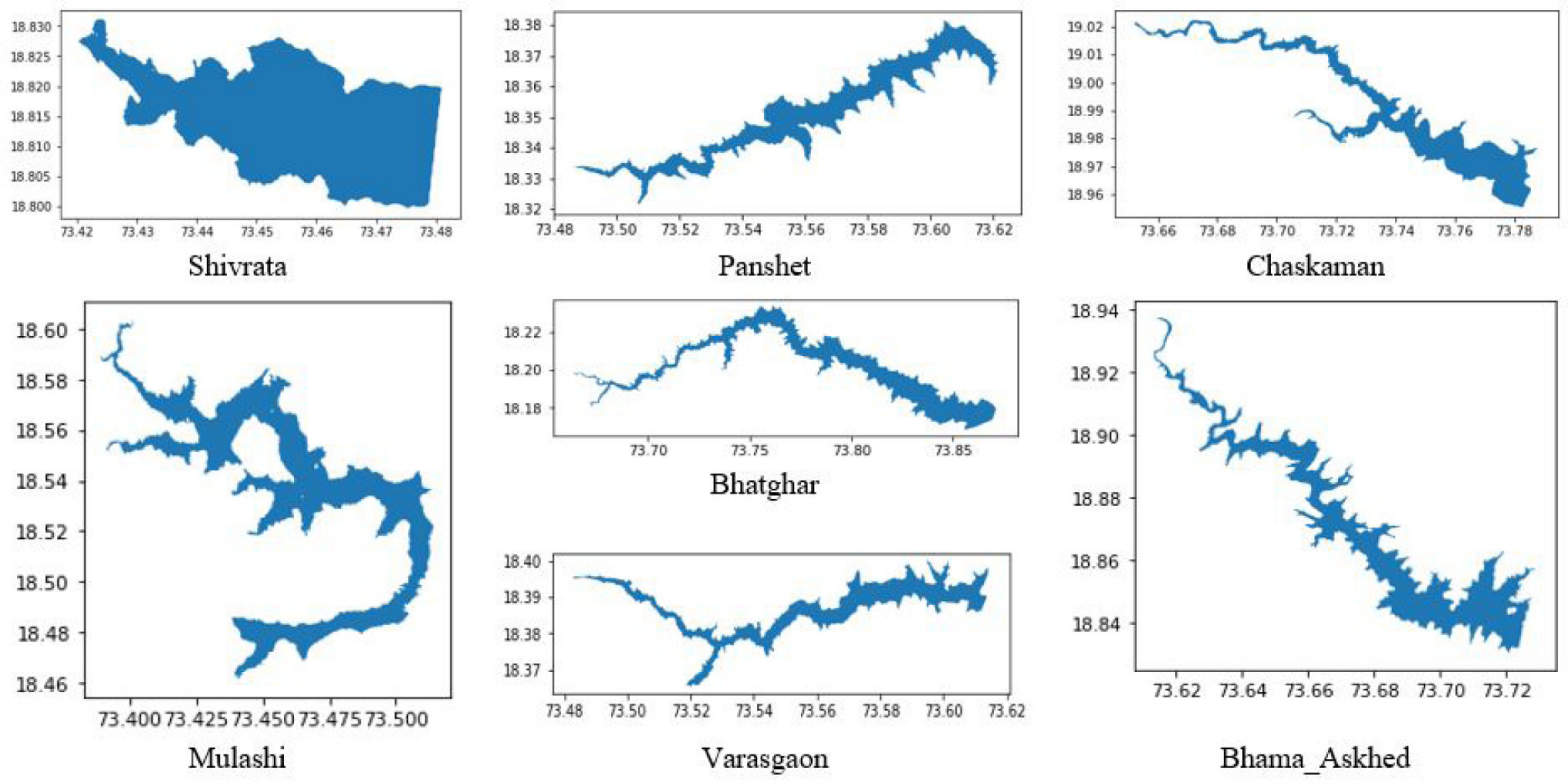
Identified reservoirs location and shape.

The intra-annual distribution of surface water is prepared by mapping maximum water extents during eight months of the year in 2016. The comprehensive picture of the dynamics of the surface water on yearly basis is prepared. The percentage of occurrence at which water was present on the surface during all months except monsoon season, June to September, was captured in a single image ( Figure 8). Performing the accuracy analysis of the map is not possible and can be compared with a similar database. The JRC GSWE map has weekly, monthly and yearly data of surface water. The annual surface water map, JRC GSWE annual image of 2016, is delineated for the study area extent ( Figure 9). The JRC GSWE yearly image is a single image representing all months of water occurrence in that year, in terms of permanent and seasonal water.

The overall water occurrence computed by MNDWI during the dry period only, would be biased because it would give more weight to the dry period than the wet period.

 This could be a limitation of the study and proposed for a future aspect of the research.

### Reservoir surface area comparison

**Figure 10.**
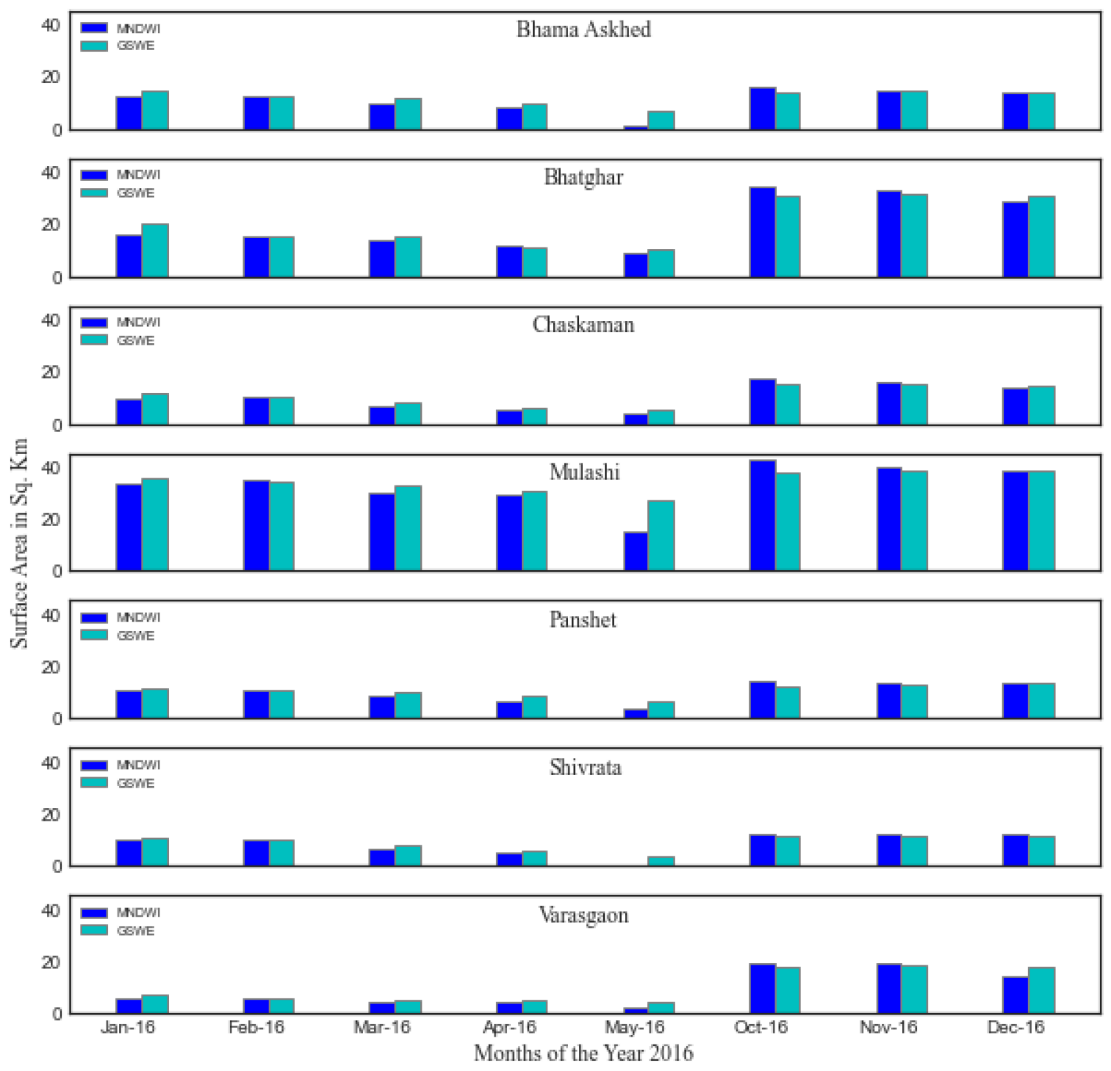
Comparative representation of surface area of distinct reservoirs in study area calculated by Modified Normalized Difference Water Index (MNDWI) and Global Surface Water Explorer (GSWE) methods.

The surface area of seven clearly visible reservoirs in the MNDWI maps and with surface area more than 500 hectors, were estimated for each of the eight months and compared to the surface area of those reservoirs calculated using GSWE data for the same months ( Table 3). The seven reservoirs are Bhama Askhed, Bhatghar, Chaskaman, Mulashi, Panshet, Shivrata and Varasgaon. The information on water occurrence and recurrence is presented in Figure 9 and Figure 10. The pattern in changing surface area by both the methods is the same. The surface area of all reservoirs is higher by GSWE methods during all the eight months except October and November, which is immediately after the monsoon months. The maximum difference in the values of surface area is observed in May and October. During May, the water storage is less in reservoirs with deteriorated water quality. In both the cases, the MNDWI method classified water for less surface area values. After the immediate monsoon, the surrounding dry land areas of the water bodies are generally misclassified as water bodies class areas due to wetness during the monsoon the in spectral indices method. The thematic maps and temporal profile were used to characterize the transition between the months of the years. MNDWI performed better in delineating large water bodies, as demonstrated by comparison to the reference data. The method also monitored the changes in quantity of water on a yearly basis.

## Conclusions

Inland surface water bodies in Pune district were detected and mapped using MNDWI spectral index derived from Landsat 8 OLI. The surface water was detected and mapped using cloud free images from eight months of the year 2016. Long term monitoring of surface water using spectral indices derived from Landsat is possible, however it is susceptible to cloud presence and some topographic restrictions.

Automatic Otsu’s threshold method is tested to separate water and non-water classes from the MNDWI image. The results obtained with Otsu’s method are not satisfactory. The MNDWI images input to Otsu’s method represent a threshold in the range -1 to 0.86. The image is multi-modal and has more than two classes. Otsu’s method requires that the number of pixels of both the classes should be proportionate. The MNDWI image has large discrepancy between the number of water and non-water pixels. The trial and error method of threshold slicing is tedious and time consuming. The trial and error method was used in iterative manner to achieve a better result. The threshold is different for all MNDWI images acquired on different dates. The Kappa coefficient represent better agreement between MNDWI and GSWE reference maps. The result obtained by trial and error methods are satisfactory.

The shadow depth derived using DEM is integrated with the MNDWI image to remove the shadow effect. The shadow effect is successfully removed and better results are achieved.

An annual surface water occurrence map is prepared from monthly surface water maps to study the dynamics of surface water. Permanent water occurrence at seven reservoirs with more than 100 Ha surface area were analyzed and compared for surface water areas on a yearly basis with reference images.

Accuracy analysis of the result is challenging because of the unavailability of physically measured data. The physically measured data for such cases is rarely available. The result obtained in this study are compared with the reference map. Hence, the comprehensive accuracy analysis could be a limitation of this study. The performance of the method may differ from the result presented in this study, by implementation of improved automatic threshold method, consideration of vegetation cover, and water quality as an aspect of further research.

##  Data availability

### Underlying data

The cloud free Landsat 8 Operational Land Imager (OLI) images of the Pune District (path:147 and row:047) that were used in this study are publicly available from the US Geological Survey (USGS) Global Visualization Viewer here: https://earthexplorer.usgs.gov/

The Joint Research Centre Global Surface Water Explorer (JRC GSWE) monthly map used for comparison in this study ^44^ is publicly available for download here: https://jeodpp.jrc.ec.europa.eu/ftp/ jrc-opendata/GSWE/MonthlyHistory/VER3-0/ tiles/2016/2016_12/

Zenodo: Surface Water Maps of Pune District in India. https://doi.org/10.5281/zenodo.6599673 ^48^. This project contains the following underlying data:

- Pune-SRTM_DEM.tiff (SRTM Digital Elevation Model representing surface topography of the study area).
- Pune-SRTM_DEM.hdr (metadata about the Pune-SRTM_DEM.tiff).
- Surface_Water_Pune_20160121.tif (binary image representing the water and non-water areas in the study area on 21st January 2016. The nomenclature of the file is as Surface_Water_Pune_YYYYMMDD. Similar files are available at the same link for the other cloud free dates in 2016).
- Surface_Water_Pune_20160121.hdr (meta-data about the image file from 21st January 2016. The nomenclature of the file is as Surface_Water_Pune_YYYYMMDD. Similar files are available at the same link for the other cloud free dates in 2016).

Zenodo: Top of Atmospheric Reflectance (ToA) Landsat 8 OLI. https://doi.org/10.5281/zenodo.6640388 ^46^.

This project contains the following underlying data:

- RT_LC08_L2SP_147047_20160121_20200907_02_ T1_SR_B1.TIF (top of atmospheric reflectance calculated from the raw digital number of Landsat 8 OLI on 21s January 2016. The image contains seven visible bands named as B1 to B7. All images captured on cloud free days of the year 2016 are made available at the same link).
- RT_LC08_L2SP_147047_20160121_20200907_02_ T1_SR_B1.TIF.aux.xml (the description of the above image in xml format. All descriptions for each image are made available at the same link).

Data are available under the terms of the Creative Commons Attribution 4.0 International license (CC-BY 4.0).

### Extended data

Analysis code available from: https://github.com/rushikulk/Otsus_Threshold/tree/v1.0.0

Archived analysis code at time of publication: https://doi.org/10.5281/zenodo.6602545 ^47^.

License: GPL-3.0-only
