# Supplementary material for: Detecting, extracting, and mapping of inland surface water using Landsat 8 Operational Land Imager: A case study of Pune district, India
Source: F1000Res. 2022 Jul 11;11:774. [Version 1] doi: 10.12688/f1000research.121740.1 (PMC9839946; doi:10.12688/f1000research.121740.1)
Supplement: Peer review history

# Peer review: Reviewer response for version 1

**DOI:** 10.5256/f1000research.133637.r143930

**Published:** 2023-01-13

**Authors:** Phaisarn Jeefoo

**Affiliations:** 1 Research Unit of Spatial Innovation Development (RUSID), Geographic Information Science, School of Information and Communication Technology (ICT), University of Phayao, Mae Ka, Thailand

Regarding this article, the article is scientifically valid in its current form. The experimental design, including controls and methods, is adequate; results are presented accurately and the conclusions are justified and supported by the data. I have comments on 4 items as follows:

 Comment 1: Increase the resolution clarity of the symbol description of figures 2, 4, 5, 9, and 10. The authors should increase the contrast of the figure more - the letters and numbers are too small and makes it difficult for the reader to see clearly.

 Comment 2: Conclusion - should add content about limitation of the study.

 Comment 3: Copyediting should be done before indexing. 

 Comment 4: References section should be re-checked again before being indexed.

Is the work clearly and accurately presented and does it cite the current literature?

Yes

If applicable, is the statistical analysis and its interpretation appropriate?

Yes

Are all the source data underlying the results available to ensure full reproducibility?

Yes

Is the study design appropriate and is the work technically sound?

Yes

Are the conclusions drawn adequately supported by the results?

Yes

Are sufficient details of methods and analysis provided to allow replication by others?

Yes

Reviewer Expertise:

Remote Sensing and GIS

I confirm that I have read this submission and believe that I have an appropriate level of expertise to confirm that it is of an acceptable scientific standard.

# Peer review: Reviewer response for version 1

**DOI:** 10.5256/f1000research.133637.r148769

**Published:** 2022-09-14

**Authors:** Bharath R

**Affiliations:** 1 Impact Forecasting, Aon Consulting Pvt., Ltd., Bangalore, Karnataka, India

Review of “Detecting, extracting, and mapping of inland surface water using Landsat 8 Operational Land Imager: A case study of Pune district, India” by Rushikesh Kulkarni, Kanchan Khare, and Humera Khanum

 The paper focuses on using established methods to detect and extract waterbodies and their applications on a case study. The originality of the work lies in the application as such a study has not been carried out for the waterbodies around city of Pune previously. This study is vital to understand the water availability and demand dynamics around the city that may eventually help town planning officials to make sound decisions in the future.  The use of open-source platforms like QGIS and GEE will enable reproducibility of the approach and thus can be applied to other parts of the country/world. 

 Comments:

1. Figure 2: The figure would need to be enhanced as it is of a lower resolution. 
2. Figure 2 is introduced before Figure 1. It is advised to change the order of the sections as Study area – Data – Methods instead of the current Methods – study area – data. This could also resolve the discontinuity, as the introduction of methods is in page 4 and the description of the methods in page 6. 
3. Figure 6 pertains to mountain shadows and Figure 7 about the Otsu method. However, in the write-up Otsu method is discussed prior to mountain shadow effect. The authors could consider changing Figure 6 to reflect Otsu Method results followed by Figure 7 about mountain shadow. This would help in improving continuity of the paper. 
4. The MNDWI index has been used to determine Annual surface water occurrence map and reservoir surface area comparison. However, there is no mention about them in the previous sections. The authors could list them as applications of their analysis in any relevant section.  

Is the work clearly and accurately presented and does it cite the current literature?

Yes

If applicable, is the statistical analysis and its interpretation appropriate?

Yes

Are all the source data underlying the results available to ensure full reproducibility?

Yes

Is the study design appropriate and is the work technically sound?

Yes

Are the conclusions drawn adequately supported by the results?

Yes

Are sufficient details of methods and analysis provided to allow replication by others?

Yes

Reviewer Expertise:

Hydrology, Flood model development, Remote sensing and GIS application in Hydrology.

I confirm that I have read this submission and believe that I have an appropriate level of expertise to confirm that it is of an acceptable scientific standard.
